# Shrub and vegetation cover predict resource selection use by an endangered species of desert lizard

**DOI:** 10.1038/s41598-020-61880-9

**Published:** 2020-03-17

**Authors:** Christopher J. Lortie, Jenna Braun, Michael Westphal, Taylor Noble, Mario Zuliani, Emmeleia Nix, Nargol Ghazian, Malory Owen, H. Scott Butterfield

**Affiliations:** 1 Department of Biology, York University, Toronto, ON Canada; 2 The National Center for Ecological Analysis and Synthesis, UCSB, Santa Barbara, CA USA; 3 The Bureau of Land Management, Marina, CA USA; 4 The Nature Conservancy, San Francisco, CA USA

**Keywords:** Conservation biology, Ecological modelling, Behavioural ecology, Ecological modelling

## Abstract

Globally, no species is exempt from the constraints associated with limited available habitat or resources, and endangered species in particular warrant critical examination. In most cases, these species are restricted to limited locations, and the relative likelihood of resource use within the space they can access is important. Using *Gambelia sila*, one of the first vertebrate species listed as endangered, we used resource selection function analysis of telemetry and remotely sensed data to identity key drivers of selected versus available locations for this species in Carrizo Plain National Monument, USA. We examined the probability of selection given different resource types. Increasing shrub cover, lower and relatively more flat sites, increasing normalized difference vegetation index, and solar radiation all significantly predicted likelihood of observed selection within the area sampled. Imagery data were also validated with fine-scale field data showing that large-scale contrasts of selection relative to available location patterns for animal species are a useful lens for potential habitat. Key environmental infrastructure such as foundation plant species including shrubs or local differences in the physical attributes were relevant to this endangered species.

## Introduction

Conservation, restoration, and rewilding of complex systems are global grand challenges. Important conceptual developments in these fields include habitat occupancy models^1^, species distribution models^2^, and home-range analyses with occurrence data^3^. We need to know how plants and animals use and occupy space. Nonetheless, many animal populations including endangered species likely have limited access to potential resources and habitats. The old adage ‘location, location, and location’ certainly applies to all species under threat because the capacity to choose to use habitat can be constrained by availability and relative location. This is a profoundly importantly perspective for strategic restoration and protection because extinction risks are predicted to rise to 30–50% globally^4^. Strategic restoration can be framed as evidence-based decision making. It is informed by science that directly shapes planning where to protect lands and identifies attributes on these lands that can function as critical tipping points. Here, we use a resource selection function analysis for a key indicator desert species regionally^5^ to provide an estimate of the relative probability of habitat selection within its home range^6^. This tool on its own does not necessarily assess the value of specific habitat features within a landscape, but it does provide a crucial stepping stone in understanding areas with greater intensities of selection by sampled individuals relative to availability^7^. We show that shrub cover is a crucial habitat type used by an endangered species of lizard in Carrizo Plain National Monument, California. Environmental predictors of relative use including elevation, slope, and solar radiation were also important and effectively described the environment with a normalized vegetation difference index. Data at many scales are important, and here we found that downscaled estimates from imagery of relevant ecological attributes of the location consistently predicted the fine-scale association with simple features for this species from field telemetry relocations. These scoping data must however be coupled with fine-scale sampling and meaningful ecological insights of interactions^8,9^. This large protected area thus likely provides critical resources for an endangered species satisfying some of its associated conservation priorities^10,11^.

**Figure 1:**
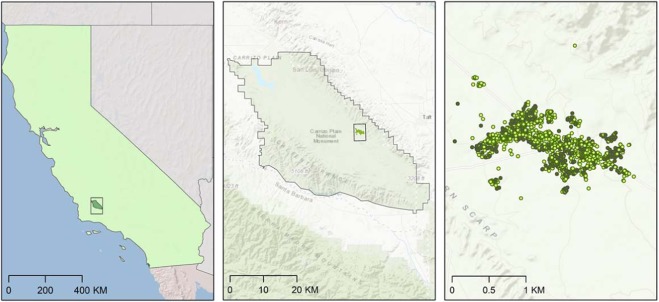
A set of maps showing three different scales of potential animal occurrence data and subsequent models for an endangered animal species case study in California. From left-to-right, the first panel shows California within an inset denoting the regional sampling focus specific to this study. Data can be sampled throughout the entire range of a species depending on its distribution and extent and are typically modeled with Maxent. The middle inset maps the estimated home ranges using 95% minimum convex polygon models for the lizard species *Gambelia sila* within Carrizo National Monument, California, USA from 2016 to 2018. The inset on right shows the fine-grain telemetry relocation data collected as points. A total of 3553 instances were sampled for a total of 80 unique individual animals. Shrub and open designations describe whether the individual animal in each was instance was within 5 m of a shrub (dark green points) or in the relative open not near the canopy of a shrub (light green points). In this study, we use resource selection functions to infer predicted likelihoods of habitat use patterns at this scale. Base map credits ESRI.

Telemetry and the tracking of animal movement is a rapidly evolving field of research for ecology, wildlife management, and conservation globally. However, the incredible leaps in the capacity to map and quite literally test new ideas ‘at scale’ are also associated with some limitations^12,13^. Matching the scale of these data to other spatial assets and applying meaningful ecological inferences to the species at hand are non-trivial decisions. Typically, relatively large-scale animal data regionally or on landscapes are modeled using ecological niche approaches and habitat occupancy models whilst finer grain animal tracking datasets are interpreted using resource selection functions^12,13^. Resource selection functions contrast ‘used’ and ‘available’ locations within a region by a set of individuals for a species. Subsequently, this tool explores the relevant landscape attribute classifications of each set to build predictions of the realized patterns of selection for the individuals examined in a study for their habitat at fine scales. Herein, we examined the latter approach for an endangered species of lizard *Gambelia sila* (*G. sila*) because it was in the first group of species listed as endangered by the USA in 1967^14^ and because it is also an excellent indicator of ecosystem health regionally in these drylands^5^. The primary goal was to examine the relative probability of selection for different resources types within a specific landscape^7^. Reptiles are particularly sensitive to global change including habitat loss, drought, and temperature increases^5^. Importantly, previous evidence showed that it is commonly associated with shrubs within this dryland ecosystem^15,16^ thereby informing a potential key biotic driver for resource selection models. Individuals of *G. sila* were sampled within Carrizo Plain National Monument to an extent of 1.92 km^2^ (Fig. 1 shows all field relocation instances, and net area occupied was conservatively derived from 95% minimum convex polygon home-range estimates, see Methods). This distribution is similar to previous wildlife studies published for *G. sila*^17,18^. This endangered species was most frequently relocated belowground, i.e. in burrows (GLM, Chi-square _mesohabitat_ = 4.61, p = 0.0001), and relocation frequencies increased with higher shrub densities estimated in the field (GLM, Chi-square _shrub density_ = 0.85, p = 0.0001). These data also included an estimate of the associated vegetation in each instance. The relevance of shrub cover and other features was tested as predictors in the resource selection function models. Resource selection functions fit to telemetry data tell us about the act of habitat selection not presence or use^7^. Telemetry follows a subset of individuals in a population, and the data represent the sequence of decisions made by these individuals. Collectively, this is a representation of behavior by habitat-type associations within a target landscape.

**Figure 2:**
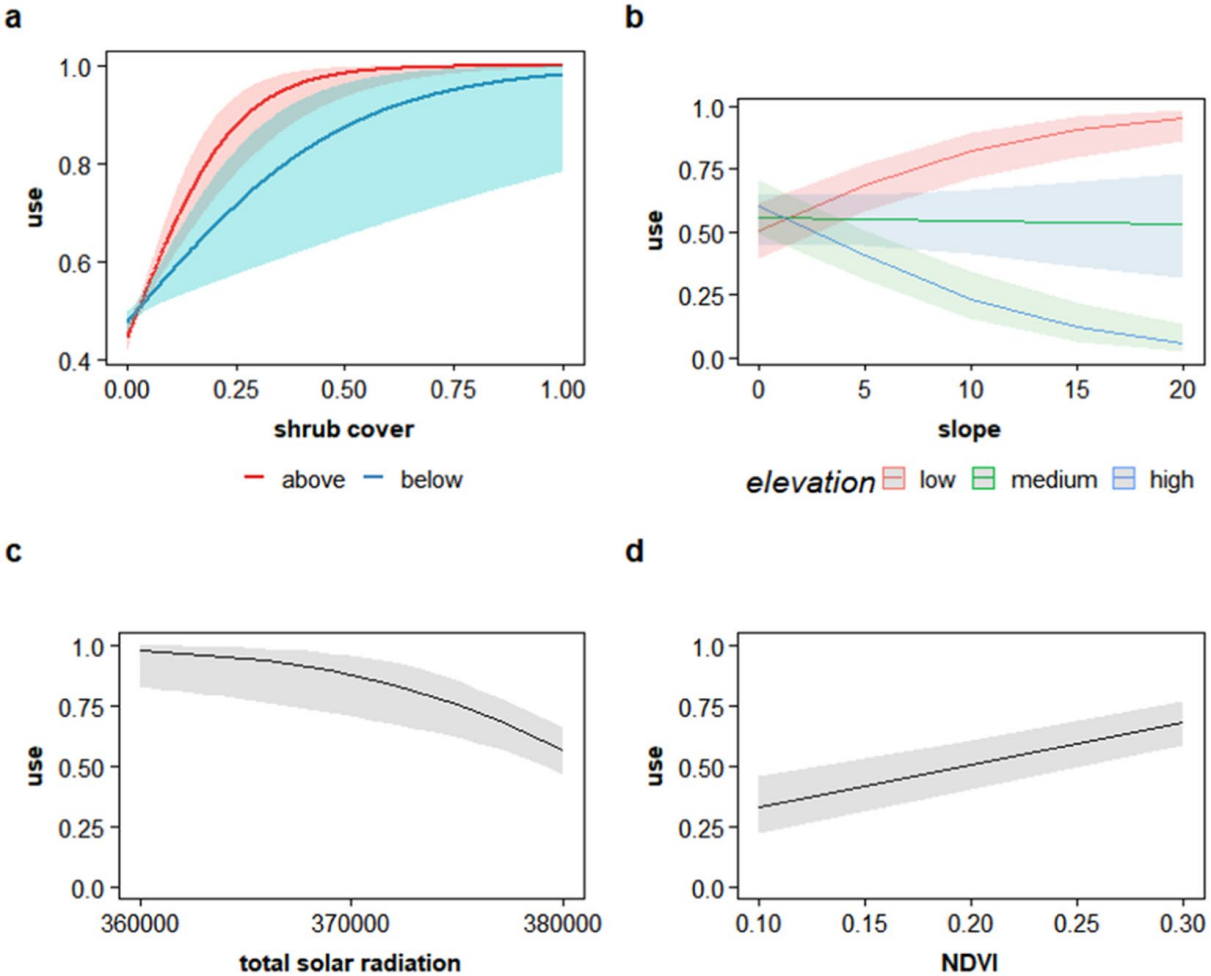
A set of resource selection function models examining the predicted probabilities of the lizard *Gambelia sila* resource use by relevant biotic and abiotic landscape attributes. Probability of use was derived from contrasts of resource selection function models for ‘used’ and ‘available’ telemetry relocation instances from a resource selection function (see Methods for full details). (**a**) Resource selection function for total probability of shrub cover estimated from imagery on the predicted probability of animal relative use. Above and below described above or belowground predicted relative use from sampled telemetry relocation field data. (**b**) The relative importance of percent slope and three elevation binned categories on the predicted relative use. Low described predicted occurrences at a mean elevation of 670 m, medium at 715 m, and high at 745 m. (**c**) Solar radiation in W/m^2^ on the resource selection function probabilities for relative use by this species. (**d**) The predicted importance of probability of normalized difference vegetation index (NDVI) on animal probability of relative use.

**Figure 3:**
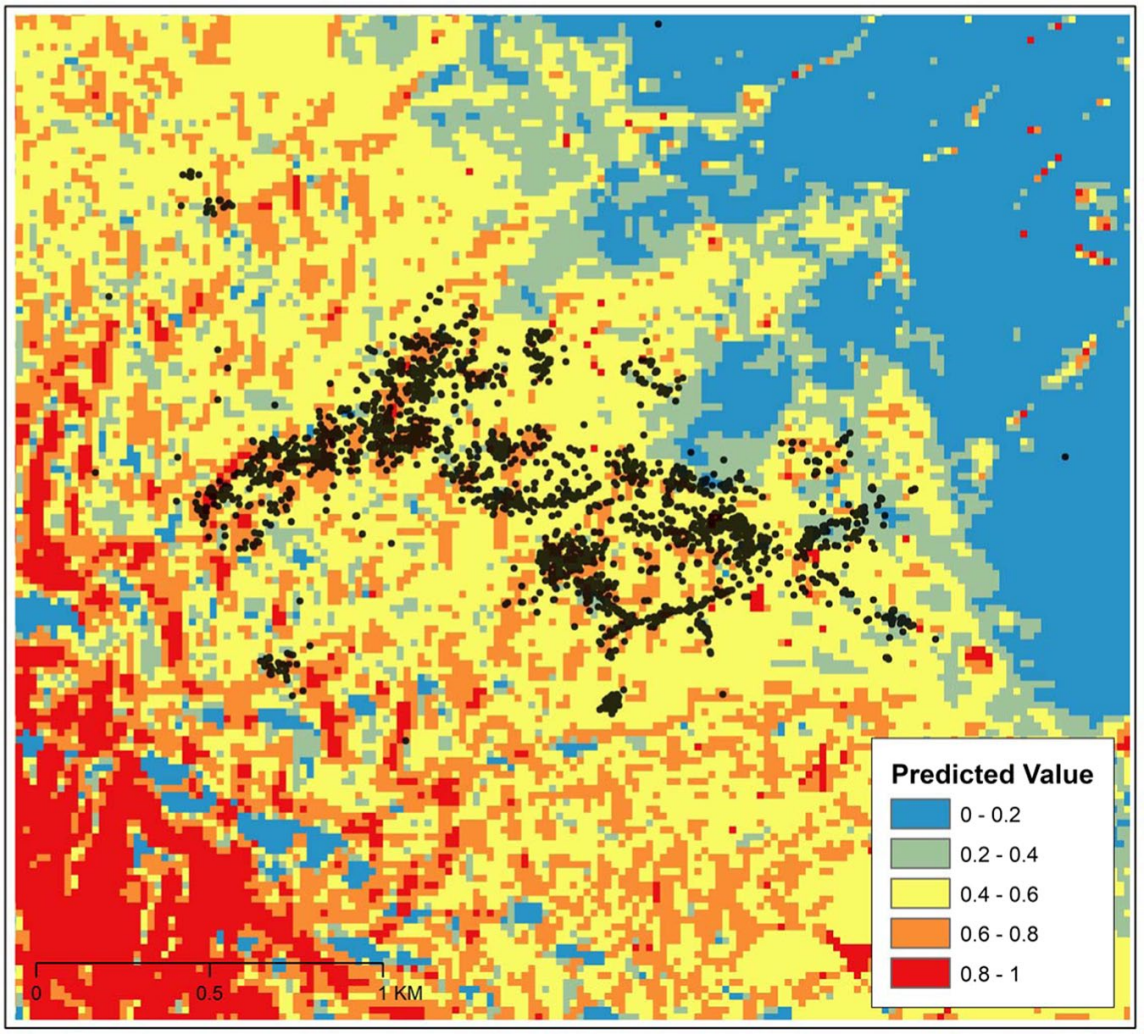
An overlay of telemetry field sampling relocation points for the lizard *Gambelia sila* on its predicted resource selection function likelihood probabilities using shrub cover, slope, aspect, elevation, and NDVI to model landscape attributes. Telemetry data were collected from 2016 to 2018 at Carrizo National Monument, USA. Blue pixels and cooler colors denote low probabilities and red denotes relatively higher estimates from the resource selection function predictive model using all factors described above.

Passive rewilding and many other restoration and analytic approaches assume dispersal, reintroductions/translocations, and availability of space are viable options. We adopt a parsimonious approach with resource selection by analyzing the importance of location relevant to a species at the scale that the animal experiences. This method ensures that we understand the patterns of selection for an indicator animal species and the preferential selection of the locations that they can realistically access. This does not provide inference to habitat value or fitness of individuals but does highlight locations with higher intensities of use hopefully for the better and not the worse because of potential ecological traps or spatial bottlenecks^19^. It has been proposed that rewilding and managing complex ecosystems must always examine the environmental infrastructure to restore an ecosystem^20^. Downscaled data from imagery for a specific study region is a mechanism to estimate these drivers. These measures can provide the potential boundaries and constraints of “location, location, and location” in addition to estimates of the relative importance of competing selection for different environmental resources^20^. We derived shrub cover, elevation, slope, solar radiation, aspect, and normalized difference vegetation index (NDVI) attributes for the landscape as simplified indirect measures for the trophic complexity (i.e. shrub cover), dispersal limitations (slope and elevation), and some stochastic processes (solar radiation) that the system experiences. We then trained models and tested the predictive capacities of these classifications for likelihood probabilities of *G. sila* presence at a protected dryland area in California, USA. These analyses were at finer grain sizes relative to previous models for this species regionally^5^. Increasing shrub cover positively predicted lizard presence (Fig. 2a, GLM, Chi-square _shrub cover_ = 41.4, p = 0.0001, Supplement A) in the resource selection function model similar to the telemetry relocation field data (described above). The effects were robust and typically consistent from year-to-year (Supplement A). Critically, for each fine-scale telemetry relocation instance, we also *in situ* coded whether the individual animal was physically associated with a dominant shrub through direct observation and used this ecological information to validate the downscaled estimates of shrub cover in subsequent models. This contrast provides a robust ground-truthing mechanism. The data aggregation approach and GIS classifications were highly representative of field sampling (GLM model cross validation, 100 model iterations, Chi-square _shrub contrasts_ = 38.57, p = 0.0001) suggesting that larger-scale data can augment finer-scale selection estimates^13^ (Supplement B). Shrub cover was more strongly selected when lizards were observed above ground similar to previous ecological research^16^. Decreasing slopes and elevations positively predicted animal presence, but at the lowest elevation class tested, increases in slope (at that elevation class) can increase the likelihood of habitat selection (Fig. 2b, GLM, Chi-square _slope_ = 188.12, p = 0.0001 & Chi-square _elevation_ = 44.31, p = 0.0001). This suggests that dispersal limitations within a region are a valid consideration for an endangered species^21^. Estimated solar radiation and aspect were significant drivers of predicted habitats confirming that stochastic processes are also relevant (Fig. 2c, GLM, Chi-square _solar_ = 3.87, p = 0.049, GLM, Chi-square _aspect_ = 138.03, p = 0.0001, Supplement C). Finally, NDVI was an important ecological predictor of habitat selection with increases in estimated total cover of all vegetation associated with higher intensities of relative selection for this endangered species (Fig. 2d, GLM, Chi-square _NDVI_ = 50.31, p = 0.0001). The effect of increases in this estimate of relative habitat intensity selection suggest that, at least at the cover levels within this ecosystem for this set of individuals, vegetation does not interfere with animal movement and either increases non-trophic complexity by providing shade or refuge from predators or indirectly enhances complexity through increased access to prey items such as invertebrates living in or under plants^16^. An overlay of telemetry sampling on the predicted resource function probability grid cells using shrub cover, slope, aspect, elevation, and NDVI (Fig. 3) suggests that vegetation including shrubs can be an important component of predicting desert vertebrate animal selection patterns in drylands. These finding support previous research showing that ecological foundation species such as shrubs in drylands consistently facilitate other species^22,23^.

Protected areas can be large-scale sandboxes for restoration architects to experiment, document, and build critical conservation plans similar to this project. Initially, these landscapes were established to protect iconic places or species such as *G. sila* but are facing increasing demands to deliver local, long-term biodiversity functions^10^. There is a call to both upsize and interconnect these critical areas but to do so adaptably depending on context and constraints^11^. Hence, we need comprehensive data on ‘bright spots’ such as Carrizo Plain National Monument and other public lands worldwide that are both iconic and functional that support and highlight a diversity of habitats^24^. Blending occurrence and species presence data with models aligns with this goal but needs to be ground-truthed through sampling such as telemetry and other animal tracking tools^25,26^. Planners must now include function for many species as a key criterion to avoid continuing declines and mismatches between protected areas and the needs that locations provide for species^10,11^. Extinct is too late, and rare and endangered species within these protected sandboxes can be indicators of change^27^. Extinction triggers can be direct or indirect, and it has been proposed that some of the drivers that precipitate species rarity by changing the structure and function of supporting ecology can also lead to future extinctions^27^. Interactions and functions can thus both lead to cascading effects on ecosystem structure^28^. The resource selection function is a proxy for habitat selection^29^, and neither this specific tool nor protected areas are new ideas^10,30^. Selectivity is a product of preference by the animals and habitat, and we need to examine these interactions quantitatively and iteratively (i.e., https://cjlortie.shinyapps.io/Resource_selection_function_app/). Resource selection behavior is also highly context dependent with behavior changing as the availability of different habitat types changes (i.e., a functional response)^6^. Critical attributes with associated likelihood estimates of contemporary selection are nonetheless critical conservation data for many animal species, and the potential interaction between preference and availability for locations is cogent to conservation and biodiversity.

## Methods

### Telemetry

At Carrizo National Monument, California, USA (35.1914°N, 119.7929°W) within the San Joaquin Desert^31^, a 5 km^2^ was identified for ecological research. This site was comprised of a foundation shrub species Mormon tea, *Ephedra californica*^15,32,33^, other mixed annual and perennial vegetation^34^, and was occupied by the blunt-nosed leopard lizard, *Gambelia sila*^5,15^. For three years from 2016–2018, up to 30 individual lizards were tracked using telemetry each summer when this species was primarily active^15^. Small VHF radio transmitters were attached to individuals (Holohil model BD‐2, frequency 151–152 MHz) and were relocated using a 3‐element Yagi antenna with a Model R‐100 telemetry receiver (Communications Specialists, Inc., Orange, CA, USA). Each instance was geotagged to within 5 m, and specific ecological elements of habitat including shrub or open canopy microsites and micro-topography were recorded. A total of 3553 relocations were recorded, and only 6 outlier relocation data points outside the 5 km^2^ study area were excluded. The study area was defined as the full extent of sampling across all three years (and the 6 excluded points were likely incorrect coding instances by field observers). At each sampling instance, the association of the individual animal was also coded as shrub or open defined as within 5 m of a visible shrub estimated using a laser rangefinder - Bushnell Outdoor Products, Overland Park, KS, USA and as above or belowground. All data are freely available at figshare (DOI: 10.6084/m9.figshare.8239667.v1). All experimental protocols for field sampling were approved by the California Department of Fish and Wildlife for *Gambelia sila*. All methods including collar deployment and removal were carried out by M. Westphal in accordance with relevant guidelines and regulations (https://nrm.dfg.ca.gov/FileHandler.ashx?DocumentID=174900&inline).

### Digital raster processing and remote sensing

ArcGIS 10.6.1 and R version 3.5.3^35^ were used for all analyses. The NAD 1983 UTM Zone 11N projection was used for the geographic manipulations. Datasets using the datum WGS 1984 were transformed to matching using WGS_1984 to NAD_1083 within ArcGIS. Shrub cover was classified at 0.6 m from the 2016 National Agriculture Imagery Program dataset using the maximum likelihood classifier algorithm in ArcGIS. Training samples were created by visually choosing shrub, open space, and shadow pixels. Pairwise scatterplots and histograms of the three imagery classes were used to ensure adequate separation of the pixels. Shrub pixels were aggregated to 20 m and divided by pixel area to represent percent shrub cover. The digital elevation model (STRM) was resampled to 20 m using bilinear interpolation. Slope and aspect were calculated using the spatial analyst extension and resampled to match the shrub cover and elevation datasets. Aspect was converted into categorical variable. The normalized difference vegetation index(NDVI) was calculated from LANDSAT 8 (April 23^rd^ 2019) using the following equation: NDVI = (NIR − RED)/(NIR + RED) and resampled to 20 m using bilinear interpolation.This single, representative values estimates peak biomass within a year and was used for all analyses. Solar radiation was calculated for each 20 m pixel of the digital elevation model using the spatial analyst extension for the months of May to July to cover peak activity periods, and the output is total radiation in watt hours per m^2^. The environmental covariates were extracted to cell centroids of the 20 m grid and spatially joined to the telemetry locations.

### Resource selection function methods

We calculated the 95% minimum convex polygon (MCP) for all points using the R package adehabitat^36^. Individual telemetry relocations that fell within the 95% MCP were classified as used locations. The MCP was modeled as equally available to all these relocation instances. For each, a fully randomized point (generated in an independent R dataframe) was then selected from the available area The used and available points can overlap in space because this does not influence the coefficient estimates but removing data points can influence range estimates^37^. We tested several used:available ratios reported in the literature (Singleton 2010, Northrup 2013) and found that 1:1 was adequate to stabilize coefficient estimates. These points were then examined using two workflows to ensure results were robust and to cross-validate interpretation^38^. First, the resource selection R package was applied as a direct, simple test of differences between used and available habitat features by this set of individuals^39^. Specifically, we used the resource selection probability function (rspf) model for presence only data with a logit link function^7,40,41^. The rspf provides probability values whilst rsf provides relative probability^7^. Secondly, we used a logistic generalized linear regression model with shrub cover, elevation, slope, NDVI, solar radiation and aspect as predictor variables (GLM). The individual lizard ID was treated as a fixed effect to account for the individual responses of lizards. We found that mixed models including the lizard ID as a random intercept or slopes were not tractable with the full range of environmental predictors we were investigating. “Recent literature suggests that including” individual identifiers as fixed effects adequately estimates the coefficients (Muff 2019).

AIC scores were calculated for models^6^, and 95% confidence intervals for the variable coefficients were computed using the confint R function. We repeated this second workflow splitting the dataset into mesohabitat (shrub vs. open) and ground use (above vs. below). As these dataset splits have fewer points than the complete relocation dataset, we sampled two available points for every used telemetry point to capture environmental variation within the study area to stabilize the coefficient estimates. To describe the potential distribution of lizard populations, we used the ‘predict’ function over a 5 km^2^ map around the study site showing a greater extent of Carrizo Plain National Monument. This is not the same as a resource selection function but is an excellent alternative mechanism to estimate likelihoods with the same key features. We also compared above vs. “below ground predicted spatial extent of the use” intensities for the 5 km^2^ study site by comparing the mean predicted values of the predicted 20 m pixels^42^. All analyses were done in R version 3.6.0, and code is publicly archived at zenodo (DOI: 10.5281/zenodo.3240619) with additional exploratory data analyses and models also available at GitHub (https://cjlortie.github.io/Carrizo_telemetry/). The simple resource selection function R package tests are available here: https://cjlortie.github.io/Resource_selection_Carrizo/.

## Supplementary information

Supplementary information

## References

[1] Bailey LL, MacKenzie DI, Nichols JD (2013). Advances and applications of occupancy models. Methods in Ecology and Evolution.

[2] Guisan A, Thuiller W (2005). Predicting species distribution: offering more than simple habitat models. Ecology Letters.

[3] Walter WD, Onorato DP, Fischer JW (2015). Is there a single best estimator? Selection of home range estimators using area-under-the-curve. Movement Ecology.

[4] Thomas CD (2004). Extinction risk from climate change. Nature.

[5] Stewart JAE (2019). Habitat restoration opportunities, climatic niche contraction, and conservation biogeography in California’s San Joaquin Desert. Plos One.

[6] Holbrook JD (2019). Functional responses in habitat selection: clarifying hypotheses and interpretations. Ecological Applications.

[7] Lele SR, Merrill EH, Keim J, Boyce MS (2013). Selection, use, choice and occupancy: clarifying concepts in resource selection studies. Journal of Animal Ecology.

[8] Papageorgiou D, Barboutis C, Kassara C, Giokas S (2017). Habitat selection of woodchat shrikes Lanius senator during spring stopover is related to foraging strategy. *Current*. Zoology.

[9] Christian, K. A., Webb, J. K. & Schultz, T. J. Energetics of bluetongue lizards (Tiliqua scincoides) in a seasonal tropical environment. *Oecologia* **136**, 10.1007/s00442-003-1301-9 (2003). 10.1007/s00442-003-1301-9 12774225

[10] Watson JEM, Dudley N, Segan DB, Hockings M (2014). The performance and potential of protected areas. Nature.

[11] Pringle RM (2017). Upgrading protected areas to conserve wild biodiversity. Nature.

[12] Morris LR, Proffitt KM, Blackburn JK (2016). Mapping resource selection functions in wildlife studies: Concerns and recommendations. Applied Geography.

[13] Hebblewhite, M. & Haydon, D. T. Distinguishing technology from biology: a critical review of the use of GPS telemetry data in ecology. *Phil. Trans. R. Soc. B.* **365**, 10.1098/rstb.2010.0087 (2010). 10.1098/rstb.2010.0087 PMC2894965 20566506

[14] Service, U. S. F. A. W. Recovery plan for upland species of the San Joaquin Valley. *Calfornia*., 1–319 (1998).

[15] Westphal MF, Noble T, Butterfield HS, Lortie CJ (2018). A test of desert shrub facilitation via radiotelemetric monitoring of a diurnal lizard. Ecology and Evolution.

[16] Filazzola A (2017). Non-trophic interactions in deserts: Facilitation, interference, and an endangered lizard species. Basic and Applied Ecology.

[17] Bailey CV, Germano DJ (2015). Probability of occurrence of Blunt-nosed Leopard Lizards on habitat patches of various sizes in the San Joaquin Desert of California. Western Wildlife.

[18] Germano DJ, Rathbun GB (2016). Home Range and Habitat Use by Blunt-nosed Leopard Lizards in the Southern San Joaquin Desert of California. Journal of Herpetology.

[19] Hale R, Swearer SE (2016). Ecological traps: current evidence and future directions. Proceedings of the Royal Society B: Biological Sciences.

[20] Perino A (2019). Rewilding complex ecosystems. Science.

[21] Halstead BJ, Wylie GD, Casazza ML (2014). Ghost of habitat past: Historic habitat affects the contemporary distribution of giant garter snakes in a modified landscape. Animal Conservation.

[22] Borst ACW (2018). Foundation species enhance food web complexity through non-trophic facilitation. Plos One.

[23] Thomsen MS (2018). Secondary foundation species enhance biodiversity. Nature Ecology & Evolution.

[24] Cooke SJ (2018). Evidence-based restoration in the Anthropocene—from acting with purpose to acting for impact. Restoration Ecology.

[25] Matzek V, Gornish ES, Hulvey KB (2017). Emerging approaches to successful ecological restoration: five imperatives to guide innovation. Restoration Ecology.

[26] Statham MJ (2019). Noninvasive Identification of Herpetofauna: Pairing Conservation Dogs and Genetic Analysis. The Journal of Wildlife Management.

[27] Hull PM, Darroch SAF, Erwin DH (2015). Rarity in mass extinctions and the future of ecosystems. Nature.

[28] Paschke, M. W., Perkins, L. B. & Veblen, K. E. Restoration for multiple use. *Restoration Ecology* **0**, 10.1111/rec.12949 (2019).

[29] Aarts G, MacKenzie M, McConnell B, Fedak M, Matthiopoulos J (2008). Estimating space-use and habitat preference from wildlife telemetry data. Ecography.

[30] Boyce MS, McDonald LL (1999). Relating populations to habitats using resource selection functions. Trends in Ecology & Evolution.

[31] Germano DJ (2011). The San Joaquin Desert of California: Ecologically Misunderstood and Overlooked. Natural Areas Journal.

[32] Lortie, C. J., Filazzola, A. & Westphal, M. In *Knowledge Network for Biocomplexity* (2017).

[33] Cutlar HC (1939). Monograph of the North American species of the genus *Ephedra*. Annals of the Missouri Botanical Garden.

[34] Lucero JE (2019). The dark side of facilitation: native shrubs facilitate exotic annuals more strongly than native annuals. NeoBiota.

[35] R-Core-Team. R: A Language and Environment for Statistical Computing. *R Foundation for Statistical Computing*, R version 3.5.3 (2019).

[36] Calenge, C. Home range estimate in R: the adehabitatHR package. *CRAN* **0.4.16** (2019).

[37] Johnson CJ, Nielsen SE, Merrill EH, McDonald TL, Boyce MS (2006). Resource Selection Functions Based on Use–Availability Data: Theoretical Motivation and Evaluation Methods. Journal of Wildlife Management.

[38] Northrup JM, Hooten MB, Anderson CR, Wittemyer G (2013). Practical guidance on characterizing availability in resource selection functions under a use–availability design. Ecology.

[39] Solymos, P. ResourceSelection: Resource Selection (Probability) Functions for Use-Availability Data. *CRAN* Version 0.3-5 (2019).

[40] Lele SR, Keim JL (2006). Weighted distributions and estimation of resource selection probability functions. Ecology.

[41] Lele SR (2009). A New Method for Estimation of Resource Selection Probability Function. The Journal of Wildlife Management.

[42] Muff S, Signer J, Fieberg J (2019). Accounting for individual-specific variation in habitat-selection studies: Efficient estimation of mixed-effects models using Bayesian or frequentist computation. Journal of Animal Ecology.

