# Supplementary material for: Shrub and vegetation cover predict resource selection use by an endangered species of desert lizard
Source: Sci Rep. 2020 Mar 17;10:4884. doi: 10.1038/s41598-020-61880-9 (PMC7078218; doi:10.1038/s41598-020-61880-9)

**Shrub and vegetation cover predict resource selection use by an endangered species of desert lizard.**

Christopher J. Lortie^1,2,*^, Jenna Braun^1^, Michael Westphal^3^, Taylor Noble^1^, Mario Zuliani^1^, , Emmeleia Nix^3^, Nargol Ghazian^1^, Malory Owen^1^, and H. Scott Butterfield^4^.

1. Department of Biology, York University, Toronto, ON, Canada.

2. The National Center for Ecological Analysis and Synthesis, UCSB, Santa Barbara, CA, USA.

3. The Bureau of Land Management, Marina, CA, USA.

4. The Nature Conservancy, San Francisco, CA, USA.

* Correspondence: lortie@yorku.ca, PH: 416.736.2100 x 20588, FAX: 416.736.5698

**Supplementary materials**

**A. A contrast of key factors from ecological telemetry sampling estimated using resource selection models. Values are from Wald test. See Methods for model fitting.**

**Mesohabitat**

|  | Open | | | Shrub | | |
| --- | --- | --- | --- | --- | --- | --- |
|  | **LR Chisq** | **Df** | **Pr(>Chisq)** | **LR Chisq** | **Df** | **Pr(>Chisq)** |
| Shrub cover | 6.390 | 1 | 0.0114 | 80.121 | 1 | 0 |
| Slope | 148.631 | 1 | 0 | 34.611 | 1 | 0 |
| Elevation | 31.730 | 1 | 0 | 7.196 | 1 | 0.0073 |
| NDVI | 41.066 | 1 | 0 | 8.537 | 1 | 0.0035 |
| Solar | 2.354 | 1 | 0.1249 | 0.872 | 1 | 0.3505 |
| Aspect | 27.992 | 8 | 0.000 | 33.772 | 8 | 0.00004 |
| Lizard ID | 19.623 | 75 | 1 | 10.246 | 67 | 1 |
| Slope:Elevation | 150.548 | 1 | 0 | 35.703 | 1 | 0 |
| Solar:Aspect | 27.819 | 8 | 0.0005 | 33.709 | 8 | 0.00005 |

**Below and above ground**

|  | Belowground | | | Aboveground | | |
| --- | --- | --- | --- | --- | --- | --- |
|  | **LR Chisq** | **Df** | **Pr(>Chisq)** | **LR Chisq** | **Df** | **Pr(>Chisq)** |
| Shrub cover | 8.352 | 1 | 0.0038 | 36.554 | 1 | 0 |
| Slope | 129.982 | 1 | 0 | 75.862 | 1 | 0 |
| Elevation | 34.611 | 1 | 0 | 11.472 | 1 | 0.0007 |
| NDVI | 24.792 | 1 | 0.0000006 | 32.480 | 1 | 0 |
| Solar | 7.567 | 1 | 0.0059 | 1.053 | 1 | 0.3048 |
| Aspect | 22.505 | 8 | 0.0041 | 40.906 | 8 | 0.000002 |
| Lizard ID | 18.461 | 76 | 1 | 9.976 | 71 | 1 |
| Slope:Elevation | 132.915 | 1 | 0 | 77.077 | 1 | 0 |
| Solar:Aspect | 22.343 | 8 | 0.0043 | 40.819 | 8 | 0.000002 |

**Study year**

|  | Year 1 | | | Year 2 | | | Year 3 | | |
| --- | --- | --- | --- | --- | --- | --- | --- | --- | --- |
|  | **Chisq** | **Df** | **P** | **Chisq** | **Df** | **P** | **Chisq** | **Df** | **P** |
| Shrub cover | 61.31 | 1 | 0 | 1.609 | 1 | 0.205 | 15.050 | 1 | 0.0001 |
| Slope | 98.938 | 1 | 0 | 33.330 | 1 | 0 | 58.206 | 1 | 0 |
| Elevation | 76.969 | 1 | 0 | 16.0512 | 1 | 0.00007 | 0.073 | 1 | 0.787 |
| NDVI | 5.0389 | 1 | 0.0248 | 62.866 | 1 | 0 | 38.587 | 1 | 0 |
| Solar | 0.697 | 1 | 0.404 | 0.711 | 1 | 0.399 | 0.908 | 1 | 0.341 |
| Aspect | 32.116 | 8 | 0.00009 | 17.421 | 8 | 0.026 | 33.824 | 8 | 0.00004 |
| Lizard ID | 7.700 | 26 | 0.999 | 6.955 | 19 | 0.994 | 24.949 | 30 | 0.727 |
| Slope:Elevation | 101.270 | 1 | 0 | 34.879 | 1 | 0 | 58.932 | 1 | 0 |
| Solar:Aspect | 31.945 | 8 | 0.0001 | 17.354 | 8 | 0.027 | 33.721 | 8 | 0.00005 |

**Global**

|  |  | | |
| --- | --- | --- | --- |
|  | **LR Chisq** | **Df** | **Pr(>Chisq)** |
| Shrub cover | 44.113 | 1 | 0 |
| Slope | 124.828 | 1 | 0 |
| Elevation | 28.009 | 1 | 0.0000001 |
| NDVI | 31.003 | 1 | 0 |
| Solar | 2.783 | 1 | 0.095 |
| Aspect | 28.458 | 8 | 0.0004 |
| Lizard ID | 35.615 | 77 | 0.999 |
| Slope:Elevation | 127.660 | 1 | 0 |
| Solar:Aspect | 28.257 | 8 | 0.0004 |

**B. The AIC model selection criteria for the global logistic resource selection model reported in text.**

| Candidate model | df | AIC |
| --- | --- | --- |
| Intercept only | 1 | 9301.263 |
| Shrub_Cov + Slope + Elev + aspect.cat + NDVI + Solar | 91 | 9,091.511 |
| Shrub_Cov + Elev * Slope + aspect.cat + NDVI + Solar | 92 | 8,948.245 |
| Shrub_Cov * Slope + Elev + NDVI + Solar | 84 | 9,164.304 |
| Slope * Elev + NDVI + Solar*Shrub_Cov + aspect.cat | 93 | 8,938.928 |
| Shrub_Cov + Slope * Elev + NDVI + Solar*aspect.cat | 100 | 8,935.987 |

**C. The 95% confidence intervals for coefficients associated with each resource selection function variables modeled.**

|  | 2.5 % | 97.5 % |  | 2.5 % | 97.5 % |
| --- | --- | --- | --- | --- | --- |
| (Intercept) | -87.229 | 261.000 | lizard780 | -0.544 | 0.707 |
| Shrub_Cov | 5.302 | 10.100 | lizard80 | -0.641 | 0.461 |
| Slope | 4.637 | 7.466 | lizard800 | -0.336 | 0.933 |
| Elev | 0.002 | 0.019 | lizard841 | -0.549 | 0.691 |
| NDVI | 4.788 | 10.094 | lizard9 | -0.859 | 0.220 |
| Solar | -0.001 | 0.0002 | lizard901 | -0.570 | 0.676 |
| aspect.catflat | -805.242 | 56.534 | lizard919 | -0.691 | 0.549 |
| aspect.catnorth | -589.822 | -130.136 | lizard93 | -0.596 | 0.502 |
| aspect.catnortheast | -717.084 | -129.921 | lizard941 | -0.342 | 0.995 |
| aspect.catnorthwest | -342.621 | 19.179 | lizard960 | -0.638 | 0.624 |
| aspect.catsouth | -346.860 | 29.402 | lizard980 | -0.583 | 0.672 |
| aspect.catsoutheast | -219.570 | 195.999 | lizard99 | -0.604 | 0.489 |
| aspect.catsouthwest | -272.750 | 85.805 | lizarda | -0.468 | 0.690 |
| aspect.catwest | -206.530 | 144.197 | lizardaa | -1.003 | 0.913 |
| lizard12 | -0.591 | 0.467 | lizardab | -0.417 | 0.690 |
| lizard12B | -0.696 | 0.382 | lizardac | -0.625 | 0.502 |
| lizard13 | -0.605 | 0.498 | lizardb | -0.177 | 0.919 |
| lizard130 | -0.350 | 0.748 | lizardc | -0.086 | 1.024 |
| lizard1337 | -0.690 | 0.413 | lizardd | -0.420 | 0.683 |
| lizard16 | -0.750 | 0.319 | lizarde | -1.292 | 1.545 |
| lizard176 | -0.887 | 0.264 | lizardf | -1.544 | 1.175 |
| lizard19 | -0.436 | 0.660 | lizardg | -0.432 | 1.331 |
| lizard2 | -0.753 | 1.688 | lizardi | -0.705 | 0.436 |
| lizard20 | -0.554 | 0.523 | lizardj | -0.581 | 0.574 |
| lizard200 | -0.686 | 0.394 | lizardk | -0.558 | 0.561 |
| lizard22 | -0.583 | 0.474 | lizardl | -0.264 | 0.868 |
| lizard23 | -0.758 | 0.416 | lizardm | -0.765 | 0.380 |
| lizard25 | -0.719 | 0.379 | lizardn | -0.476 | 0.646 |
| lizard26 | -0.688 | 0.513 | lizardo | -0.266 | 0.851 |
| lizard30 | -0.596 | 0.506 | lizardp | -0.373 | 0.730 |
| lizard31 | -0.518 | 0.575 | lizardr | -0.448 | 0.664 |
| lizard35 | -0.730 | 0.727 | lizards | -0.574 | 0.542 |
| lizard381 | -0.357 | 0.892 | lizardt | -0.776 | 0.596 |
| lizard39 | -0.517 | 0.557 | lizardu | -0.648 | 0.461 |
| lizard4 | -0.473 | 0.615 | lizardv | -0.177 | 0.950 |
| lizard40 | -0.616 | 0.533 | lizardw | -0.640 | 0.499 |
| lizard420 | -3.464 | 3.059 | lizardx | -2.027 | 1.508 |
| lizard439 | -0.504 | 0.777 | lizardy | -1.984 | 6.044 |
| lizard44 | -0.572 | 0.507 | lizardz | -0.479 | 0.660 |
| lizard460 | -0.657 | 0.611 | lizard740 | -0.740 | 0.515 |
| lizard462 | -3.526 | 2.992 | lizard760 | -0.502 | 0.742 |
| lizard47 | -0.565 | 0.551 | Slope:Elev | -0.010 | -0.007 |
| lizard480 | -0.631 | 0.626 | Solar:aspect.catflat | -0.0001 | 0.002 |
| lizard540 | -0.727 | 0.536 | Solar:aspect.catnorth | 0.0003 | 0.002 |
| lizard56 | -0.614 | 0.472 | Solar:aspect.catnortheast | 0.0003 | 0.002 |
| lizard620 | -0.482 | 0.790 | Solar:aspect.catnorthwest | -0.0001 | 0.001 |
| lizard64 | -0.423 | 0.664 | Solar:aspect.catsouth | -0.0001 | 0.001 |
| lizard640 | -0.332 | 0.918 | Solar:aspect.catsoutheast | -0.001 | 0.001 |
| lizard680 | -3.255 | 3.313 | Solar:aspect.catsouthwest | -0.0002 | 0.001 |
| lizard7 | -0.660 | 0.421 | Solar:aspect.catwest | -0.0004 | 0.001 |

**D. Home-range models derived from 95% minimum convex polygons. See Methods for details**.


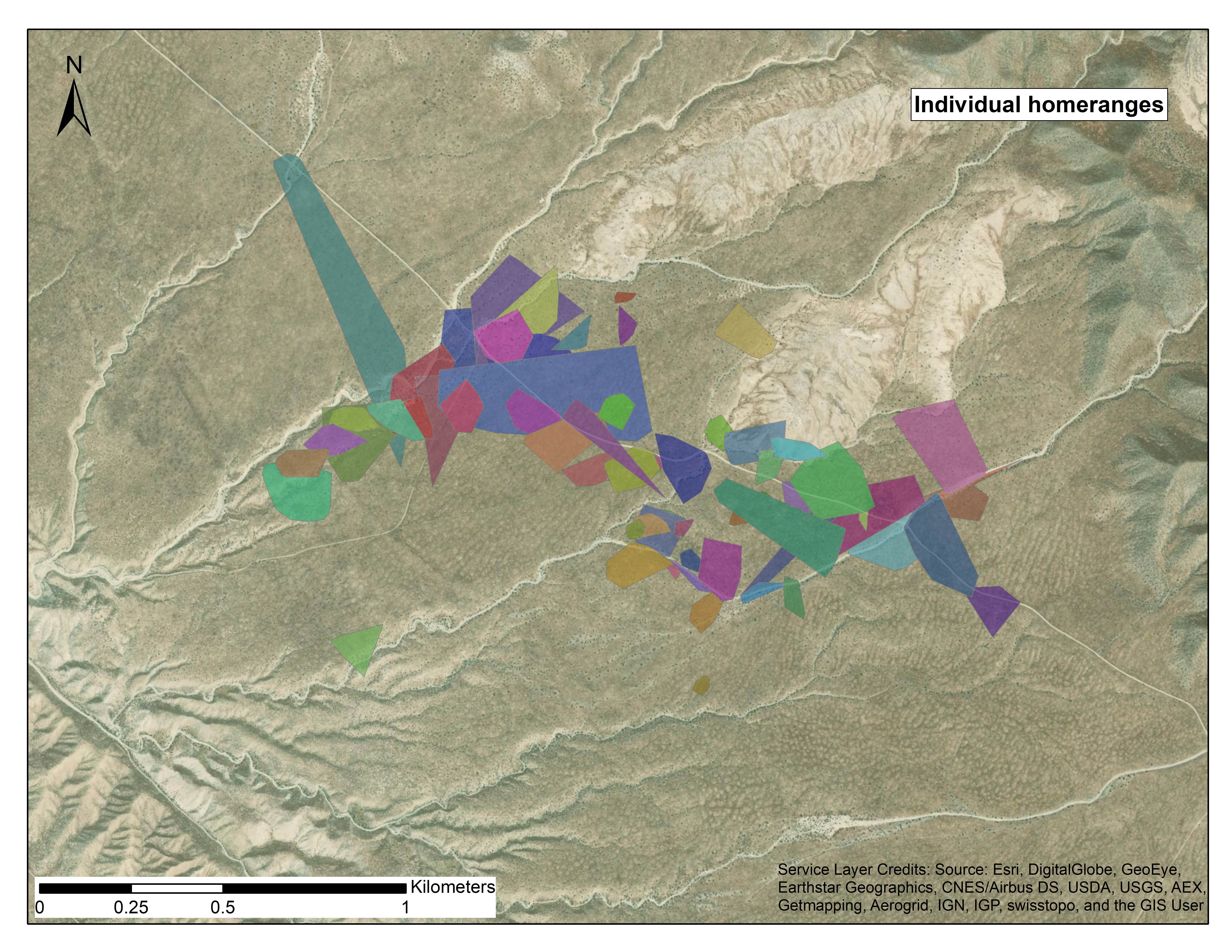

Supplement: Supplementary file 1 — Supplementary information [file 41598_2020_61880_MOESM1_ESM.docx]
